# Demographic determinants of breast self-examination among adult females in Botswana

**DOI:** 10.4102/curationis.v49i1.2848

**Published:** 2026-06-30

**Authors:** Punishment P. Chibatamoto, Chester Kalinda, Moses J. Chimbari

**Affiliations:** 1 School of Medicine, College of Health Sciences, University of KwaZulu-Natal, Durban, South Africa; 2 Bill and Joyce Cummings Institute of Global Health, University of GLobal Health Equity (UGHE), Kigali, Rwanda

**Keywords:** Botswana, females, breast cancer, breast self-examination, practice, early diagnosis

## Abstract

**Background:**

Breast cancer is a major public health problem in Botswana, with an age-adjusted incidence of 20 per 100 000 females in 2020. It is the second most common female cancer. Between 72% and 90% of patients present with advanced stages at first diagnosis, which severely impacts survival. Breast self-examination (BSE) provides an easy, low-cost and safe tool to improve early detection.

**Objectives:**

This study aimed to determine the prevalence of and factors influencing BSE uptake among females across Botswana.

**Method:**

A cross-sectional hospital-based study was conducted from September 2023 to January 2024 among 158 females aged at least 18 years across four hospitals in Botswana. Systemic random sampling was used. Data on sociodemographic factors and BSE practices were collected via a pretested, Setswana-translated questionnaire adapted from the Breast Cancer Initiative. Analysis via StataNow 18 SE utilised descriptive statistics, Chi-square tests and binary logistic regression. Ethical approval and written consent were obtained.

**Results:**

Most participants (77.8%, (*n* = 123) reported performing BSE, but only 10.6% (*n* = 13) practised it monthly as required. Only 27.8% (*n* = 44) had ever undergone a clinical breast examination. Multivariate analysis identified a lack of religious affiliation (adjusted odds ratio [AOR] 0.14; 95% confidence interval [CI] 0.03–0.63, *p* = 0.011) and lower education (AOR 0.12; 95% CI 0.03–0.56, *p* = 0.011) as significant predictors of decreased BSE practice. Conversely, females aged 30 years – 49 years were more likely to practise BSE than those aged 18 years – 29 years (AOR 3.14; 95% CI 1.22–8.03, *p* = 0.017).

**Conclusion:**

Most females in Botswana do not perform BSE regularly.

**Contribution:**

This study highlights three interactive predictors (religion, education and age) for BSE practice. We recommend integrating BSE education into school curricula, community outreach and healthcare provider training to improve breast health outcomes.

## Introduction

Breast cancer is the second most diagnosed cancer among females globally (Bray et al. 2024), with an estimated 2.3 million new cases and 670 000 related deaths globally (Arnold et al. 2022). It is projected that globally, one in 20 females will be diagnosed with breast cancer in their lifetime, resulting in an estimated 3.2 million new breast cancer cases and 1.1 million breast cancer-related deaths annually by 2050 (Kim et al. 2025). In sub-Saharan Africa (SSA), the incidence and mortality of breast cancer have been on the rise, and in the absence of interventions, they have been projected to double by 2050 (Pace & Shulman 2016). According to the Global Cancer Observatory, SSA accounted for 1 198 300 new cases and 91 300 deaths related to breast cancer in 2022 (Fu et al. 2025). Breast cancer places a substantial burden on the SSA region, with incidence rates per 100 000 females ranging from a high of 46.2 in Southern Africa to a low of 27.9 in Central Africa, and associated mortality rates are high, though mortality rates vary across the regions (Sung et al. 2021). Sung et al. (2021) further indicate that the high burden of cancer in SSA is exacerbated by low 5-year survival rates, estimated at 40%.

The survival rates of breast cancer in SSA are poor, with 79% at 1 year after diagnosis, 56% at 3 years and 40% at 5 years after diagnosis (Limenih et al. 2024). In African countries with comprehensive data, such as South Africa, Cameroon, the Central African Republic, Malawi and Tanzania, most cases were presented late for diagnosis, defined as a delay of 3 months or more from the onset of symptoms to the first clinical consultation (Kohler et al. 2017). Breast cancer among females is a major public health problem in Botswana, with an age-adjusted incidence of 20 per 100 000 females (Sung et al. 2021). It is the second most common cancer diagnosed in females after cervical cancer. While previous studies suggested that between 72% and 90% of Botswana females presented with advanced-stage breast cancer at diagnosis (Bhatia et al. 2019; Mbuka-Ongona & Tumbo 2013), a 2025 study found a lower, yet still significant, prevalence of 46% (*n* = 90) at the initial diagnosis stage diagnosis (Chibatamoto, Kalinda & Chimbari 2025).

The high prevalence of late diagnoses contributes to reduced survival rates and increased mortality, underscoring the importance of enhanced access to effective diagnostic services and timely treatment (Barrios 2022). The poor survival rates for females with breast cancer in SSA are associated with late-stage presentation because of both system and patient-related factors, including low awareness, absence of organised early detection programmes, inadequately trained healthcare workers, unavailability and inefficient screening services, inaccessibility of treatment and insufficiently equipped health facilities for accurate and timely diagnosis and treatment (Gebremedhin & Shamebo 1998; Kene et al. 2010; Mabula et al. 2012; Winters, Mannell & Esser 1988)

According to the World Health Organization (WHO 2020), early diagnosis is the cornerstone of breast cancer management. Hence, health-seeking behaviour should be promoted for early screening and linkage to appropriate treatment for improved survival. Health-seeking behaviour is associated with good uptake of breast cancer screening methods, including breast self-examination (BSE), clinical breast examination (CBE) and mammography (Omidiji et al. 2017). These breast screening methods play a crucial role in early disease detection and prevention (Albeshan et al. 2020). Mammography, a low-dose X-ray of the breast, is more effective at detecting breast cancers early, particularly those that are not yet palpable and can find changes years before physical symptoms develop. However, in resource-constrained countries like Botswana, implementing breast examinations by healthcare professionals has been challenging as a result of a combination of factors related to training, resources and cultural contexts (Ba et al. 2020; Pierz & Randall 2020; Sarker et al. 2022). These challenges often result in missed diagnoses and delayed treatment, hindering efforts to improve breast cancer outcomes.

Although mammography is the gold standard for breast cancer diagnosis, CBE and BSE are considered valuable for breast awareness and as adjuncts to mammography (Farrell, Bennett & Schwartz 2020). Clinical breast examination is a physical examination of the breasts and surrounding areas performed by a trained healthcare professional, and BSE is a technique where females examine their own breasts for any changes or abnormalities (Shockney 2023). Though not as effective in reducing mortality as mammography, CBE and BSE can help females become familiar with their breasts and potentially detect abnormalities that might be missed by mammography alone (Shah & Guraya 2017). Thus, regular practice of BSE empowers females to make informed decisions about screening practices and lifestyle choices for improved breast cancer outcomes.

Effective breast cancer management relies on women’s consistent practice of BSE, driven by their belief in its importance, not just healthcare availability. Despite Botswana’s high geographic healthcare accessibility (90% of its 2.36 million population live within 5 km – 8 km of a facility), a 2019 Ministry of Health (MOH) and WHO study indicates that this does not necessarily guarantee access to quality care (Statistics Botswana 2022a; World Health Organization Regional Office for Africa 2022). Furthermore, a significant gap exists in the literature regarding BSE prevalence and uptake across Botswana, as the few existing studies are mainly confined to Gaborone, the capital city of Botswana. One study conducted in 2011 on females attending a Gaborone health facility found that while most participants (63.5%) had practised BSE at some point, only a minority (23.5%) did so monthly as recommended (Tiengo, Pengpid & Peltzer 2011).

Another study exploring the Health Belief Model identified that only 10.4% of Botswana female students screened for breast cancer and access to health professionals for information was significantly associated with uptake (Tapera et al. 2019). While these studies provided valuable information, a significant amount of time has passed since then, and the health landscape, public awareness campaigns and socioeconomic factors have all changed. Furthermore, regional disparities in Botswana may exist regarding BSE knowledge and practice. A study conducted in one area, such as Gaborone, may not be representative of the entire country, as indicated by data from a Botswana Harvard Partnership report that shows significant variation in CBE rates across different towns (Dykstra et al. 2021).

Lack of comprehensive, up-to-date, actionable information on BSE prevalence and its determinants remains a significant hurdle in breast cancer prevention efforts in Botswana. Therefore, this study determined the prevalence of and the factors influencing the uptake of BSE among females across Botswana. The findings from this study will inform the development of culturally appropriate and effective health promotion campaigns to improve early detection rates.

## Research methods and design

### Study design

We conducted a cross-sectional hospital-based study among females seeking health services in outpatient departments at the four primary hospitals located in the northern (Francistown district), southern (Goodhope district), northwestern (Maun district) and central (Serowe district) parts of Botswana.

### Sampling

The study population consisted of all adult females exiting outpatient departments at all four designated public health cancer centres. The sample size was calculated via the following formula (Equation 1):

$$n=t^{2}\text{pq}/d^{2}$$ [Eqn 1]

where *n* = minimum sample size needed; *t* = 1.96 value of confidence level desired; *p* = 10% prevalence of correct BSE performance determined in a pre-testing exercise at one non-participating hospital; and *d* = 5% precision. Based on these parameters, the calculated effective sample size was 139. Assuming a non-response rate of 10%, the final sample size was computed to be 153 participants.

### Data collection

A team of experienced data collectors was trained to conduct exit interviews using a Setswana (local language)-translated questionnaire adapted from the Breast Cancer Initiative (Dvaladze et al. 2016). Before its full application, the questionnaire was pre-tested with a sample of 20 females at Princess Marina Referral Hospital, in Gaborone (non-participating health facility) to assess its validity, reliability, content validity and internal consistency. This pre-test also yielded a 10% crude prevalence of correctly performing BSE among the participants. The results of the pre-test validated the questionnaire’s ability to accurately and consistently capture the intended responses to breast cancer questions. Data collected during this preliminary phase were excluded from the main study analysis.

After the pre-test, the questionnaire was administered to adult females (18 years and above) exiting outpatient consultation rooms between 15 September 2023 and 15 January 2024 at each of the four participating public health facilities. Further, at each participating health facility, systematic random sampling was applied in selecting every third client exiting the outpatient consultation room until the calculated sample size of 153 was exceeded. The participants eligible for the study included adult females aged 18 years and above with no history of breast cancer or any other cancers, not pregnant or breastfeeding, citizens of Botswana and seeking health services at the outpatient departments of the participating hospitals. Exclusion criteria included psychiatric conditions, active substance abuse or the inability to communicate in either Setswana or English. The questionnaire obtained information on participants’ sociodemographic characteristics, BSE and CBE practice.

### Data analysis

Data analysis was conducted using StataNow 18 SE. Descriptive statistics were used to summarise the sociodemographic factors, perceptions and attitudes of the study participants towards breast cancer screening. Bivariate analysis was performed using the chi-square test to explore the relationship between sociodemographic factors and BSE uptake. In instances where the expected cell count was less than five in more than 20% of the cells, Fisher’s exact test was applied instead. Subsequently, a binary logistic regression model was used for multivariate analysis to identify independent predictors of BSE practice while controlling for potential confounders.

### Ethical considerations

Ethical approval was obtained from the Health Research Development Committee (HRDC) of the MOH, Botswana, with ethical application reference number HPDME 13/18/1, as well as South Africa, ethical application reference number University of KwaZulu-Natal (UKZN) Biomedical Research Ethics Committee (BREC) (No. BREC/00001596/2020). Written informed consent was obtained from all participants prior to their involvement. All research was conducted in accordance with the principles laid out in the Declaration of Helsinki.

## Results

### Participants’ characteristics

One hundred and fifty-eight adult females participated in this study; 32.9% (*n* = 52) were in Maun, 28.5% (*n* = 45) in Goodhope, 22.8% (*n* = 36) in Francistown and 15.8% (*n* = 25) in Serowe. Their mean age (± s.d.) was 38.71 (± 13.20) years. Most participants were single (71.5%, *n* = 113. A smaller proportion were either married (17.7%, *n* = 28), cohabiting (3.8%, *n* = 6), divorced (3.2%, *n* = 5) or widowed (2.5%, *n* = 4). Almost two-thirds (69.0%, *n* = 109) of the participants were unemployed. The remaining participants were split between formal employment (15.8%, *n* = 25) and informal employment (15.2%, *n* = 24).

Additionally, the largest segment of study participants (46.8%, *n* = 74) chose not to disclose their religious beliefs. Among those who reported an affiliation, Christians made up the largest group at 36.7% (*n* = 58), including both Pentecostal (19.6%, *n* = 31) and Protestant (17.1%, *n* = 27) adherents. A smaller, but notable, group (16.5%, *n* = 26) indicated no religious affiliation. Regarding residential distance, the results were evenly split between those living 1 km – 5 km away and those living more than 5 km away, with both groups accounting for 29.7% (*n* = 47) of respondents. Uncertainty about distance was reported by 19.6% (*n* = 31), while the remaining respondents lived less than 1 km (11.4%, *n* = 18) or exactly 1 km away (8.9%, *n* = 14).

### Prevalence of practising breast self-examination and clinical breast examination

Most of the females interviewed (81.0%, *n* = 128) said they made their own medical care decisions. In contrast, very few females consulted with their husbands (3.8%, *n* = 6) or mothers (8.2%, *n* = 13). While most respondents (77.8%, *n* = 123) reported having ever performed BSE, only 10.6% (*n* = 13) of them did so monthly as is recommended. Among the 110 (89.4%) females who did not perform BSE at the recommended frequency, the habits were inconsistent: almost half (49.6%, *n* = 61) performed BSE occasionally, while a significant number did so daily (26.8%, *n* = 33) or weekly (13.0%, *n* = 16). In addition, just about a quarter (27.8%, *n* = 44) of all survey participants had ever received a CBE.

### Factors associated with practising breast self-examination

A Chi-square test of independence was conducted to examine the relationship between BSE practice and demographic factors. The results, presented in Table 1, indicate a statistically significant association between BSE practice and religion (*p* = 0.010), marital status (*p* = 0.023) and education level (*p* = 0.017).

**TABLE 1:** Factors associated with breast self-examination practice among females in Botswana (*N* = 157).

Characteristic	Performing BSE (*N* = 122)		Not performing BSE (*N* = 35)		*p*-value
	*n*	%	*n*	%	
**Age (years)**	-	-	-	-	0.056
18–29	28	22.95	15	42.86	-
30–49	68	55.74	13	37.14	-
≥ 50	26	21.31	7	20.00	-
**Distance to nearest health facility (km)**	-	-	-	-	0.606
< 1	14	11.38	4	11.43	-
1	10	8.13	4	11.43	-
1–5	37	30.08	10	28.57	-
> 5	35	28.46	12	17.14	-
Not sure	26	21.46	5	31.43	-
**Time taken to the nearest health facility (min)**	-	-	-	-	0.858
< 30	73	59.84	20	58.82	-
30–60	25	20.49	6	17.65	-
≥ 60	24	19.67	8	23.53	-
**Religion**	-	-	-	-	0.010*
Protestant	24	19.51	3	8.57	-
Pentecostal	26	21.14	5	14.29	-
Unspecified religion	59	47.97	15	42.86	-
Not affiliated to any religion	14	11.38	12	34.29	-
**Marital status**	-	-	-	-	0.023*
Married	25	20.33	3	8.57	-
Cohabitation	3	2.44	3	8.57	-
Divorced	4	3.25	1	2.86	-
Single	87	70.75	26	74.29	-
Widowed	4	3.25	0	0.00	-
Others	0	0.00	2	5.71	-
**Level of education**	-	-	-	-	0.017*
Tertiary	25	20.33	4	11.43	-
Secondary	29	23.58	5	14.29	-
Primary	58	47.15	16	45.71	-
None	11	8.94	10	28.57	-
**Occupation**	-	-	-	-	0.496
Formal	21	17.07	4	11.43	-
Informal	20	16.26	4	11.43	-
Unemployed	82	66.67	27	77.14	-

BSE, breast self-examination; km, kilometer; min, minutes.

* , Significant (*p* < 0.05).

### Predictors of practising breast self-examination among respondents

The results of the multivariable logistic regression analysis, presented in Table 2, identify significant sociodemographic predictors of BSE practice. Age emerged as the strongest positive predictor, exhibiting a clear age-dependent gradient. Compared to the reference group (18 years – 29 years), females aged 30 years – 49 years were 2.15 times more likely to perform BSE (Odds ratio [OR] = 2.15), while those aged 50 years and older demonstrated a 3.85-fold increase in the odds of practice (OR = 3.85). Participants with primary or no education (OR = 0.28) and those with secondary education (OR = 0.42) were significantly less likely to engage in BSE compared to those with tertiary education. Additionally, religious affiliation was identified as a significant cultural factor; specifically, individuals with no religious affiliation showed significantly lower odds of practising BSE compared to Protestants. In contrast, marital status (married, cohabitating, divorced or widowed and single) was not a significant predictor of BSE practice.

**TABLE 2:** Logistic regression analysis of factors associated with breast self-examination practice (*N* = 157).

Variable	Categories	Odds Ratio	95% CI	*p*-value
Religion	Protestant (Ref)	1.00	-	-
	Pentecostal	0.61	0.28–1.35	0.224
	Unspecified	0.45	0.19–1.08	0.075
	Not affiliated	0.29	0.11–0.78	0.014*
Marital status	Married (Ref)	1.00	-	-
	Cohabitation	1.15	0.40–3.28	0.790
	Divorced or widowed	0.95	0.35–2.58	0.920
	Single	0.88	0.38–2.03	0.765
Education	Tertiary (Ref)	1.00	-	-
	Secondary	0.42	0.19–0.93	0.033*
	Primary or none	0.28	0.12–0.66	0.003*
Age category	18–29 years (Ref)	1.00	-	-
	30–49 years	2.15	1.12–4.12	0.022*
	> 50 years	3.85	1.45–10.22	0.007*

CI, confidence interval; Ref, reference.

* , significant (*p* < 0.05).

A multivariable logistic regression was further performed to understand the relationship between multiple independent variables (religion, marital status, education level and age) and BSE practice (whether a female practices BSE or not). As shown in Table 3, significant predictors of BSE practice were identified, with lack of religious affiliation (OR = 0.17; 95% Confidence Interval [CI] 0.03–0.82; *p* = 0.028) and no formal education (OR = 0.09; 95% CI: 0.02–0.47; *p* = 0.005) associated with significantly lower odds of engagement. Conversely, females aged 30 years – 49 years demonstrated a higher likelihood of practising BSE (OR = 2.95; 95% CI: 1.10–7.88; *p* = 0.031) compared to the 18 years – 29 years age group, while marital status showed no statistically significant relationship with BSE behaviour.

**TABLE 3:** Multivariable logistic regression analysis of factors associated with breast self-examination practice (*N* = 157).

Variable	Categories	OR	CI	*p*-value
Religion	Protestant (Ref)	1.00	-	-
	Pentecostal	0.51	0.10–2.70	0.429
	Unspecified	0.46	0.11–2.04	0.309
	Not affiliated	0.17	0.03–0.82	0.028*
Marital status	Married (Ref)	1.00	-	-
	Cohabiting	0.45	0.04–5.06	0.520
	Divorced or widowed or other†	0.27	0.02–3.74	0.332
	Single	0.55	0.13–2.34	0.418
Education level	Tertiary (Ref)	1.00	-	-
	Secondary	0.76	0.15–3.84	0.738
	Primary	0.39	0.10–1.57	0.185
	None	0.09	0.02–0.47	0.005*
Age (years)	18–29 years (Ref)	1.00	-	-
	30–49 years	2.95	1.10–7.88	0.031*
	> 50 years	3.04	0.70–13.16	0.137

OR, odds ratio; CI, confidence interval; Ref, reference.

* , significant (*p* < 0.05).

† , grouped because of low frequency.

As shown in Table 4, the reduced multivariate logistic regression model (*N* = 157) indicates that BSE practice is significantly lower among females with no religious affiliation compared to Protestants (Adjusted Odds Ratio [AOR] 0.14; 95% CI 0.03–0.63, *p* = 0.011) and those with primary or no education compared to tertiary education (AOR 0.12; 95% CI 0.03–0.56, *p* = 0.011). Conversely, females aged 30 years – 49 years are more likely to practice BSE compared to those aged 18–29 (AOR 3.14; 95% CI 1.22–8.03, *p* = 0.017). Data indicate that 30 years – 49 years age group, specific education levels and lack of religious affiliation are key predictors for BSE practice.

**TABLE 4:** Reduced multivariate logistic regression model (adjusted odds ratio) for breast self-examination practice (*N* = 157).

Variable	Categories	AOR†	95% CI	*p*-value
Religion	Protestant (Ref)	1.00	-	-
	Pentecostal	0.47	0.09–2.44	0.372
	Unspecified	0.39	0.10–1.59	0.198
	Not affiliated	0.14	0.03–0.63	0.011*
Education level	Tertiary (Ref)	1.00	-	-
	Secondary	0.99	0.19–1.59	0.372
	Primary or none	0.12	0.03–0.56	0.011*
Age (years)	18–29 (Ref)	1.00	-	-
	30–49 years	3.14	1.22–8.03	0.017*
	> 50 years	2.95	0.84–10.34	0.090

AOR, adjusted odds ratio; CI, confidence interval; Ref, reference.

* , significant (*p* < 0.05).

† , Adjusted odds ratio for religion, education and age.

## Discussion

In resource-limited settings, such as Botswana, where advanced breast cancer screening may not be readily available, BSE is vital in empowering females to recognise changes in their breasts, promoting breast health awareness and encouraging early medical evaluation (Bonsu & Ncama 2018; WHO 2007). Additionally, BSE screening methods are generally considered easy to perform, feasible, convenient and safe, requiring no specialised equipment or setup (Secginli & Nahcivan 2006). This study contributes to the existing body of literature by examining the factors that influence the uptake of BSE screening and proposing interventions to improve service utilisation among females in Botswana.

Our study reveals a high lifetime prevalence of BSE practice at 77.8% (*n* =123). However, this is limited by the fact that only 10.6% of these females performed the examination at the recommended monthly frequency. Nearly half (49.6%) practised BSE only occasionally. The findings reveal a pronounced ‘awareness-action gap’ regarding BSE in Botswana. While a high lifetime prevalence (77.8%) indicates broad awareness, the sharp decline to a 10.6% monthly adherence rate suggests that knowledge has not translated into routine health behaviour of detecting early warning signs like lumps or skin changes, critical for early cancer diagnosis (WHO 2025). This inconsistency presents a significant clinical risk. Irregular participation in BSE as part of breast cancer screening (49.6%) reported in this study presents a substantial risk of delayed diagnosis. As a result of the disease’s fast-paced progression, this discrepancy increases the probability that malignancies will be detected at later, less treatable stages. Consequently, current screening behaviours appear insufficient to significantly impact breast cancer mortality rates within the country.

Comparisons with other national, regional and international studies indicate a concerning downward trend. The current 10.6% adherence rate is substantially lower than the 37% reported in Gaborone in 2011 (Tiengo et al. 2011), suggesting that previous public health momentum has stalled or that new socioeconomic barriers have emerged. This performance also falls below the SSA average of 17.9% (Seifu & Mekonen 2021) and lags significantly behind international peers, with Vietnam at 15.2% (Tuyen et al. 2019), Poland at 56.7% (Woynarowska-Sołdan et al. 2019), Malaysia at 41% (Dahlui et al. 2011) and Russia at 32% (Levshin, Fedichkina & Droggachih 1998). Such underperformance in BSE practice in Botswana highlights the need for tailored, culturally specific interventions rather than generic awareness campaigns.

Furthermore, in our study, only 27.8% of participants had ever undergone a CBE, a figure consistent with that observed across other SSA nations (Burkina Faso, Cote d’Ivoire, Ghana, Kenya, Mozambique, Senegal and Tanzania), ranging from a low of 7.3% in Tanzania to a high of 31.1% in Burkina Faso (Ayebeng et al. 2025). Our findings indicate a critically low professional screening uptake of 27.8% CBE among females, leaving a vulnerable population without adequate diagnostic protection. Because both self-directed (BSE) and clinical checks (CBE) are infrequent, there is an urgent need to shift policy towards the integration of CBE into routine primary care visits. It is critical for the country to prioritise habit formation through reminder systems, such as SMS prompts, and empower healthcare providers to perform opportunistic clinical screenings to compensate for low self-screening consistency.

The findings of this study highlight critical sociodemographic determinants influencing BSE practices. Specifically, age, education level and religious affiliation emerged as significant predictors of whether females engage in this essential screening behaviour. The data reveal that females aged 30 years – 49 years are over three times more likely to practice BSE compared to the younger 18 years – 29 years age group (AOR 3.14). This disparity may suggest that as females mature, their perceived susceptibility to breast cancer increases, prompting more proactive health monitoring. Conversely, the lower engagement among younger females indicates a potential ‘optimism bias’, where younger individuals may feel they are not at risk, highlighting a need for early-intervention education.

While this finding is significant because it identifies a potential area for targeted health interventions in Botswana, global data on age and BSE practice remain mixed. For instance, while some studies in Malaysia, Ghana and India show higher uptake among females under 30 years because of better digital literacy (Al-Naggar et al. 2011; Dadzi & Adam 2019; Gupta 2009), others in Bangladesh, Cameroon, Ethiopia and Iran show an upward trend as females age, likely as a result of increased healthcare interaction (Azemfac et al. 2019; Hussen, Juhar & Mohammed 2023; Jafari et al. 2025; Tithi et al. 2018). Conversely, some studies found no age-related correlation (Al-Gburi & Alwan 2019; Champion 1992). These disparities suggest that interventions should not be ‘one-size-fits-all’ but instead be tailored to the specific age group and context. Younger females may benefit from integrating BSE education into educational curricula and promoting early detection, while older females require targeted outreach from healthcare professionals to address knowledge gaps.

Education also emerged as a vital predictor for BSE practice in our study and remains a powerful gateway to health literacy in Botswana. Females with primary or no formal education were significantly less likely to practice BSE compared to those with tertiary education (AOR 0.12). This suggests that higher education likely provides better access to health information, a stronger understanding of preventive care and higher confidence in performing self-checks. Although the estimated literacy rate for females in Botswana is high at 87.5% (Statistics Botswana 2022b) ironically, the rate of practising BSE is low. This suggests that general literacy does not automatically translate to health literacy. Barriers such as fear, cultural norms and a low perception of risk must be addressed by integrating specific breast health education into school curricula and community programmes (Yilmaz, Sayin & Oner Cengiz 2017). Our findings are in line with research from Ethiopia, Turkey and Nigeria (Bilge & Keskin 2014; Olaogun et al. 2017; Segni et al. 2016; Yazew, Alemu & Walle 2024).

Finally, religious affiliation was found to play a role in practising BSE in our study. Females with no religious affiliation were much less likely to practice BSE than Protestants (AOR 0.14). This suggests that religious institutions may serve as vital social networks for health communication or that faith-based communities provide a sense of agency and self-care that translates into preventive health actions. This mirrors findings in German and Ghana, where religious participation correlated with higher preventive screening rates (Kretzler, König & Hajek 2020; Osei-Afriyie et al. 2021). However, the relationship is complex. For instance, some studies show higher uptake among Christians compared to Muslims or those with no affiliation, while others suggest that social factors and health beliefs are more dominant predictors than religion itself (Gyedu et al. 2018; Marandu et al. 2024). Although some studies correlate religion with BSE practice, other health beliefs and social factors are consistently the primary predictors. This underscores the need for a multifaceted approach to health promotion that respects and leverages diverse social and cultural contexts.

### Strengths and limitations

A primary strength of this study was the employment of structured questionnaires adapted from the Breast Cancer Initiative (Dvaladze et al. 2016). The use of these validated instruments ensured methodological rigour and facilitated the comparability of our findings with existing global literature. Furthermore, the multi-district scope of the research provided a broad perspective on health-seeking behaviours across diverse geographical areas. While patient exit interviews provide timely, high-quality data, they inherently exclude individuals who do not utilise formal healthcare services, which may limit the findings’ generalisability to the wider community. However, this limitation is mitigated by the high coverage of the healthcare system in Botswana, where over 90% of the population lives within a 5 km – 10 km radius of a health facility, encouraging extensive utilisation of formal care. Therefore, while the findings are hospital based, they likely reflect the experiences of a broad segment of the community that regularly utilises the formal health system.

### Implications and recommendations

Our study highlights that specific demographic factors influence BSE practices in Botswana. Public health programmes should target interventions to address the unique challenges faced by less educated females and those without religious affiliations for improved breast cancer screening rates and early detection. While BSE may not be the primary tool for reducing breast cancer morbidity and mortality, it remains a valuable part of a broader breast prevention strategy, especially in low-resource settings such as Botswana. Our findings offer a clear roadmap for tailoring the National Cancer Programme and community health outreach, with the public health sector encouraged to leverage these study results to take the following actions:

- **Targeted youth outreach:** Public health campaigns in Botswana should move beyond general awareness and specifically target young females (18 years – 29 years), perhaps through social media and tertiary education institution-based wellness programmes. There is a need to demystify BSE for the youth, establishing it as a lifelong habit rather than a response to ageing.
- **Addressing the education gap:** To achieve health equity, the MOH should prioritise low-literacy health education materials. Using visual aids, radio broadcasts in Setswana and community ‘*Kgotla*’ [court] meetings can help bridge the gap for females who have not reached tertiary education levels, ensuring that BSE techniques are understood regardless of formal schooling.
- **Faith-based partnerships:** Given the influence of religion on health behaviour shown in this study, Botswana’s health promoters should collaborate more closely with local churches and religious leaders. As many Batswana are active in religious circles, these platforms are ideal for disseminating health information and normalising breast health discussions in a culturally sensitive manner.
- **Decentralised screening support:** For those with no religious or social affiliations, the role of Botswana’s Community Health Workers and Health Education Assistants becomes even more critical. Home-to-home visits may be the only way to reach isolated females who fall through the cracks of organised social or religious groups.

## Conclusion

In this study, we determined the prevalence of BSE and factors associated with its increased uptake among females who participated in this study. Additionally, the study revealed that most females did not regularly perform BSE, even though the practice is low cost and can facilitate early detection of breast cancer. This suggests a gap between the perceived importance of BSE and its actual practice. This study also offers insight into the three interactive factors (religion, education and age) among females in Botswana. The country can improve uptake of regular BSE practices by addressing the barriers that prevent females from adopting this simple, cost-effective screening technique. These findings highlight the need for increased targeted education and awareness campaigns to promote regular BSE practice. We recommend that the Government of Botswana consider integrating BSE education into school curricula, community outreach programmes and healthcare provider training. This would ultimately promote early detection of breast cancer and contribute to improved health outcomes for females in Botswana.
